# Performance, digestibility, antioxidant activity, immunity, and zinc bioavailability benefits of advanced zinc chelate supplementation in broiler diets: A comparative study

**DOI:** 10.1016/j.psj.2025.105972

**Published:** 2025-10-18

**Authors:** Mousa Nasiri, Hossein Ali Ghasemi, Iman Hajkhodadadi, Mohammad Hassan Nazaran

**Affiliations:** a Department of Animal Science, Faculty of Agriculture and Environment, Arak University, Arak, 38156-8-8349, Iran; b Department of Research and Development, Sodour Ahrar Shargh Company, Tehran, Iran

**Keywords:** Broiler chicken, Advanced zinc chelate supplement, Growth performance, Metabolic indices, Immunity

## Abstract

This study aimed to investigate the effects of various organic sources of zinc at different levels on growth performance, physiological indices, antioxidant status, immunity, and mineral metabolism in broiler chickens. A total of 1,400 one-day-old male chicks were used in a completely randomized design with 10 experimental treatments, each arranged in 5 replicate pens with 28 birds per pen. The treatments included a control diet containing zinc sulfate (ZnSO₄); zinc–methionine supplementation at levels of 40, 80, and 120 mg/kg diet (Zn-Met40, Zn-Met80, and Zn-Met120); zinc–glycine supplementation at similar levels (Zn-Gly40, Zn-Gly80, and Zn-Gly120); and advanced zinc chelate supplementation at corresponding levels (Zn-ACT40, Zn-ACT80, and Zn-ACT120). The results showed that the Zn-ACT80, Zn-ACT120, and Zn-Met80 treatments significantly increased body weight gain, improved feed conversion ratio, and increased performance index compared with the control and Zn-Gly40 treatments (*P* < 0.05). The Zn-ACT80, Zn-ACT120, Zn-Met80, and Zn-Met120 treatments significantly increased the activity of antioxidant enzymes, including superoxide dismutase and catalase, and increased total serum antioxidant capacity (*P* < 0.05). Improvements in nutrient absorption and ileal digestion of crude protein were also observed in these treatments. The Zn-ACT80 and Zn-Met120 treatments further increased zinc concentrations in the bone and pancreas and decreased zinc excretion through feces (*P* < 0.05), indicating higher bioavailability than Zn-Gly at comparable levels. An increase in the number of goblet cells and an improvement in the absorptive surface of the intestinal villi were also reported in the Zn-ACT80 and Zn-Met80 treatments (*P* < 0.05). From an economic point of view, Zn-ACT80 achieved effectiveness equivalent to, or greater than, that of Zn-ACT120 in many indices. In conclusion, the findings showed that the advanced zinc chelate was comparable to zinc–methionine in terms of bioavailability and effectiveness in improving the performance and health of broilers, and both supplements were superior to zinc–glycine, suggesting the use of advanced zinc chelate as a new and effective source of zinc in poultry diets.

## Introduction

Zinc is a trace element essential for numerous physiological functions in chickens, including immune regulation, protection against oxidative stress, and enzymatic activity (Yogesh et al., 2013; Jarosz et al., 2017). According to established nutritional standards for optimal growth and health performance (Aviagen, 2022), the dietary zinc requirement for modern broilers is typically set at 120 mg/kg. However, the bioavailability of zinc from natural feed ingredients is often limited by dietary antagonists such as phytates and high calcium levels, which impair intestinal absorption (Naz et al., 2016; Jarosz et al., 2017). Zinc oxide and zinc sulfate are two commonly used inorganic zinc sources that have long been included in poultry diets. However, their low bioavailability results in poor absorption and substantial mineral losses, which contribute to environmental pollution (Min et al., 2019; Diao et al., 2021). In recent years, organic zinc complexes have received increased attention due to their improved absorption efficiency and greater gastrointestinal stability, particularly those chelated with amino acids such as glycine or methionine (Akhavan-Salamat and Ghasemi, 2019; Niknia et al., 2022; Zhu et al., 2022). Although the findings are promising, there is ongoing debate about which organic zinc sources are most effective. Differences in chelation strength, structural stability, and molecular size may help explain the variability observed across studies (Wu et al., 2019; Kumar et al., 2021; Hu et al., 2024). A more accurate assessment of zinc bioavailability can be achieved by measuring zinc retention in specific tissues such as blood, liver, bone, and pancreas (Mwangi et al., 2017; Azad et al., 2020). Tissue mineral concentrations are considered more reliable indicators of zinc utilization than growth performance metrics alone, which are often influenced by various dietary and environmental factors (Chen et al., 2022).

Recent innovations in supplement formulation have led to the development of a novel type of zinc chelate using Advanced Chelate Compounds Technology (ACCT). These compounds are produced through a polymerization-driven self-assembly process in which organic acids selectively bind to metal ions, forming highly stable, pH-resistant chelated structures (Seyfori et al., 2018; Ghasemi et al., 2020, 2022). Bonzachicken and Bonzaplex are two ACCT-based supplements that are currently protected by U.S. and European patents (Nazaran, 2010). Experimental studies have shown that these products significantly enhance antioxidant capacity, reduce mineral excretion, and improve mineral digestibility compared to conventional organic and inorganic zinc sources (Seyfori et al., 2018; Ghasemi et al., 2020). Their improved effectiveness is attributed to structural homogeneity, enhanced compatibility with biological systems, and increased intestinal absorption due to reduced interaction with dietary antagonists (Ghasemi et al., 2023; Mohammadi et al., 2024, 2025).

Despite these promising advancements, comprehensive evaluations of ACCT-based zinc chelate at varying supplementation levels in broiler chickens remain limited. No studies have directly compared their efficacy with that of conventional organic sources, such as zinc chelated with methionine or glycine, in terms of immune response, antioxidant status, tissue mineral deposition, and overall performance. Therefore, the objective of this study was to investigate the effects of different inclusion levels of a novel zinc chelate supplement formulated using ACCT on growth performance, health status, antioxidant activity, immune function, and tissue zinc retention in broiler chickens. This supplement was compared with standard inorganic (zinc sulfate) and organic (methionine- and glycine-bound) zinc sources to assess its relative bioefficacy and potential as an improved alternative in poultry nutrition.

## Materials and methods

### Experimental design and rearing conditions

Throughout the experiment, hygiene protocols, consistent husbandry practices, and ethical guidelines for animal care and use in research were strictly followed and approved by the Animal Care and Use Committee at Arak University (approval number 1401-8687). A total of 1,400 one-day-old male broiler chickens (Ross 308), with a uniform initial body weight of 42 ± 1.1 g, were allocated in a completely randomized design (CRD) to 10 experimental treatments. The control group received a basal diet containing zinc sulfate (ZnSO₄). The remaining nine treatments included zinc–methionine supplementation at 40, 80, and 120 mg Zn/kg diet (Zn-Met40, Zn-Met80, Zn-Met120); zinc–glycine supplementation at corresponding levels (Zn-Gly40, Zn-Gly80, Zn-Gly120); and advanced zinc chelate supplementation at the same levels (Zn-ACT40, Zn-ACT80, Zn-ACT120).

In the control treatment, zinc sulfate (ZnSO₄·5H₂O) contained 34 % elemental zinc. The zinc–glycine complex (Zn-Gly) contained 26 % zinc and 36 % glycine, whereas the zinc–methionine complex (Zn-Met) contained 18.8 % zinc and 21 % methionine. The zinc chelate BonzaZinc was formulated to contain 16 % elemental zinc chelated with organic acids. The product was developed using chelation technology, in which organic acids are polymerized under controlled conditions to form a stable chelate structure. BonzaZinc, produced by Sodour Ahrar Shargh Knowledge-based Company (Tehran, Iran), was based on patented technology (US8288587B2; Nazaran, 2010).

Each treatment consisted of 5 replicates, with 28 birds per replicate. The number of replicate pens per treatment was determined a priori using a fixed-effects one-way analysis of variance model with ten treatments, a two-sided alpha of 0.05 and desired power of 0.80. Chickens were raised in a controlled environment on straw litter. Each pen measured 2.25 × 1.75 m, and all birds had ad libitum access to water and mash feed. Basal diets were formulated in three phases: starter (days 0 to 10), grower (days 11 to 24), and finisher (days 25 to 42), based on corn and soybean meal. Diets were formulated to meet metabolizable energy and nutrient requirements according to the Ross 308 strain feeding guide and differed only in the type and level of zinc supplementation (Table 1). The basal diet was prepared using a zinc-free premix to meet the requirements of Ross 308 (Aviagen, 2022), and the tested zinc supplements replaced building sand in the diet. Representative samples of each feed batch were analyzed for nutrient composition using AOAC (2007) methods. The experimental treatments by zinc sources and levels, along with the corresponding analyzed zinc concentrations, are presented in Table 2. The lighting schedule consisted of 23 hours of light and 1 hour of darkness from day 3 onward. The temperature was initially set at 33 to 34°C and decreased by 3°C per week until reaching approximately 21 to 22°C at the end of the trial.

**Table 1:** Composition of starter, grower, and finisher diets on an as-fed basis (g/kg, unless otherwise stated).

	Starter (d 0 to 10)	Grower (d 11 to 24)	Finisher (d 25 to 42)
Items			
Corn grain	545.1	601.7	642.7
Soybean meal, 44 %	331.4	256.9	220.5
Corn gluten meal, 60 %	45.1	66.5	55.2
Soybean oil	25.0	25.0	35.0
Monocalcium phosphate	14.8	13.3	11.8
Limestone	15.8	14.6	13.5
Salt (NaCl)	2.5	1.7	1.6
Sodium bicarbonate	0.6	1.8	1.9
Vitamin premix^1^	2.5	2.5	2.5
Mineral premix^2^			
L-Lysine.HCL	4.3	4.5	4.1
DL-Methionine	2.8	2.2	2.1
L-Threonine	2.0	1.6	1.4
L-Valine	0.6	0.2	0.2
Building sand 3	5	5	5
Total	1000	1000	1000
Calculated nutritive value			
Metabolizable energy, MJ/kg	12.56	12.98	13.40
Crude protein	230.0	215.0	175.5
Crude fat	49.6	51.7	63.4
Calcium	9.6	8.7	7.9
Available phosphorus	4.8	4.4	4.0
Digestible lysine	12.8	11.5	10.3
Digestible methionine	6.2	5.5	5.4
Digestible TSAA^4^	9.5	8.7	8.0
Digestible threonine	8.6	7.7	6.9
Digestible valine	9.6	8.7	7.8
Digestible tryptophan	1.9	1.7	1.5
Digestible arginine	12.7	11.2	10.1
Digestible isoleucine	8.2	7.5	6.8
Digestible leucine	13.7	12.3	11.0
Sodium	1.6	1.6	1.6
Potassium	9.5	8.1	7.4
Chlorine	2.8	2.4	2.2
DEB^5^, mEq/100 g	250	230	215
Zn, mg/kg	32	30	29

1 Supplied per kg diet: 18 mg retinol, 4 mg cholecalciferol, 36 mg a-tocopherol acetate, 2 mg vitamin K3, 1.75 mg vitamin B1, 6.6 mg vitamin B2, 9.8 mg niacin, 29.65 mg pantothenic acid, 2.94 mg vitamin B6, 1 mg folic acid, 0.015 mg vitamin B12, 0.1 mg biotin, 250 mg choline chloride and 1 mg ethoxyquin.

2 The supply of mineral elements, with the exception of zinc (which was included at different levels according to treatment), was the same across all diets, and the following amounts were provided per kilogram of diet: Mn (manganese oxide), 120 mg; Fe (ferrous sulfate), 20 mg; Cu (cupric sulfate), 16 mg; I (potassium iodide), 1.25 mg; Se (sodium selenite), 0.30 mg.

3 Dietary mineral supplements, with different Zn sources and levels (according to treatment), were added to aliquots of the basal diet at the expense of an inert filler (building sand).

4 Total sulfur amino acids.

5 DEB (dietary electrolyte balance) = (Na^+^, mEq/kg + *K*^+^, mEq/kg) – CL^−^, mEq/kg.

**Table 2:** Experimental treatments (zinc source × level) and corresponding analyzed zinc concentrations.

Treatment	Zinc source	Zinc level (mg/kg)	Description	Analyzed zinc value (mg/kg)
1	Zn sulfate	120	Control (inorganic Zn source)	146
2	Zn-Met	40	Zinc–methionine, low level	74
3	Zn-Met	80	Zinc–methionine, medium level	108
4	Zn-Met	120	Zinc–methionine, high level	148
5	Zn-Gly	40	Zinc–glycine, low level	72
6	Zn-Gly	80	Zinc–glycine, medium level	105
7	Zn-Gly	120	Zinc–glycine, high level	144
8	Zn-ACT	40	Advanced zinc chelate, low level	73
9	Zn-ACT	80	Advanced zinc chelate, medium level	112
10	Zn-ACT	120	Advanced zinc chelate, high level	150

### Measurement of growth performance and slaughter traits

To evaluate growth performance, the body weight of chicks was measured at the beginning of the period (day 0) and subsequently on days 10, 24, and 42. The body weight gain (BWG) and feed intake (FI) for each period (days 0–10, 11–24, 25–42, and the entire period 0–42) were determined and feed conversion ratio (FCR) was then calculated by dividing FI by the corresponding BWG during each period. All feed intake and weight gain data were corrected for mortality. Daily mortality was recorded and the European performance index (EPI) was calculated using the following formula:

EPI=[livability(%)×liveweight(42days,kg)×100]/[age(days)×FCR]

At the end of the experimental period (day 42), two male chickens per replicate, selected based on body weights closest to the group average, were slaughtered and necropsied. Following evisceration, the relative weights of the immune organs (spleen, bursa of Fabricius, and thymus) were recorded and expressed as percentages of the live body weight prior to slaughter.

### Digestibility assay

To assess the apparent digestibility of nutrients, 0.3 % chromium oxide (Cr₂O₃) was added to the birds’ diet from days 37 to 42 as an indigestible marker. On day 42, three birds were randomly selected from each replicate, and after slaughter, samples of the ileal contents (from the end of the jejunum to the ileocecal valve) were collected, pooled, and prepared for analysis. The samples were dried at 55–60°C, ground, and passed through a 1 mm sieve. Chemical analyses of feed and ileal samples were performed according to the AOAC methods (2011). Chromium concentration in the feed and ileal samples was determined according to Mueller (1956). Energy content was determined by evaluating dried samples using a bomb calorimeter (Parr Instrument Company, Moline, IL). Apparent ileal digestibility (AID) of dry matter, crude protein, crude fat, ash, energy, calcium, and phosphorus was then calculated based on marker and nutrient ratios using the following formula:

AID(%)=[1−(markerindigesta×nutrientindiet)/(markerindiet×nutrientindigesta)]×100

### Blood antioxidant parameters

At the end of the experiment (day 42), blood samples were collected from the wing veins of chickens and transferred into tubes. The tubes were then centrifuged to separate serum from blood cells. Malondialdehyde (MDA) levels were quantified using the thiobarbituric acid reactive substances (TBARS) method according to the method described by Jensen et al. (1997), total antioxidant capacity (TAC) was assessed by the ABTS method using commercial assay kits (Pars Azmoon, Tehran, Iran). The activities of antioxidant enzymes, including glutathione peroxidase (GPx), superoxide dismutase (SOD), and catalase (CAT), were measured using commercial assay kits (Cayman Chemical Co., Ann Arbor, MI, USA) and colorimetric enzymatic methods.

All assays were performed in duplicate with predefined acceptance criteria. For MDA measured by the TBARS method at 532 nm, the intra-assay coefficient of variation was 0.9–1.2 %, and the inter-assay coefficient of variation was 3.5–4.9 %, with a minimum detectable concentration of 0.022 µM. Total antioxidant capacity measured by the ABTS method in Trolox equivalents was linear up to 2.5 mM; within-run precision was 2.65–3.77 %, and between-run precision was 2.33–5.65 %. Enzyme assay precision from the commercial kits was as follows: GPx, intra-assay 5.5 % and inter-assay 7.0 %; SOD, intra-assay 3.1 % and inter-assay 3.5 %, with an analytical sensitivity of 0.005 units per mL; CAT, intra-assay 3.6 % and inter-assay 8.4 %, with a limit of detection of 2 nmol per min per mL.

### Measurement of zinc concentration in tibia bone, pancreas, liver, and excreta

At the end of the experimental period (day 42), 2 birds per replicate were humanely slaughtered, and tibiae were collected. The bones were defatted in ethanol using a Soxhlet apparatus for 24 h, then dried at 100°C for 24 h. After drying, the bones were incinerated in a muffle furnace at 600°C for 24 h to determine ash content. The resulting ash was dissolved in a mixture of nitric and perchloric acids, and zinc was measured by inductively coupled plasma optical emission spectrometry (ICP-OES; Optima 7000 DV, PerkinElmer, Waltham, MA, USA). To quantify zinc storage in the pancreas and liver, samples were washed, dried at 80°C, and ashed. Excreta were collected from each pen using trays over a 24 h period on day 41. Non-excreta material was carefully removed, and the pooled excreta from each pen were weighed and dried at 105°C for 48 h. After drying, the samples were ground to a fine powder and stored in sealed plastic bags. Zinc concentrations in tissues (liver, pancreas) and excreta were determined after ashing by ICP-OES.

### Measurement of antibody response against newcastle and infectious bronchitis vaccines

Vaccination followed standard veterinary guidelines for Newcastle disease virus (NDV) and infectious bronchitis virus (IBV). NDV vaccination used the Clone 30 strain (Intervet, UK) administered via drinking water on days 10 and 21 (Ghasemi et al., 2014). IBV vaccination used the Nobilis IB 4/91 strain (Intervet, UK), administered by aerosol on day 5 and via drinking water on day 21 (Omidi et al., 2015). To assess antibody responses to NDV and IBV, blood was collected from the brachial vein on days 28 and 35 (7 and 14 days after the last vaccination). Serum was separated and stored at −20°C for further analysis. Anti-NDV antibodies were determined by the hemagglutination inhibition test (Iritani et al., 1991). Anti-IBV antibodies were measured by enzyme-linked immunosorbent assay (ELISA) following Snyder et al. (1984).

### Statistical analysis

Two types of statistical designs were used to analyze the data. First, to investigate the effects of different zinc sources, a CRD with 10 treatments, including a control treatment and nine treatments representing combinations of 3 zinc sources at 3 different levels each, was employed. The means of the 10 treatments were compared using one-way analysis of variance (ANOVA), and pairwise differences were evaluated using Tukey's multiple comparison test. Prior to analysis, data normality was verified using the Shapiro-Wilk test. To investigate the interaction effects of zinc source and zinc level, a factorial design with nine treatments, comprising 3 zinc sources (zinc–methionine, zinc–glycine, advanced zinc chelate) and 3 zinc levels (40, 80, and 120 mg/kg diet) was used. Two-way ANOVA was performed to evaluate the main and interaction effects of zinc source and level on the measured response variables and means were compared using Tukey’s test. All statistical analyses were conducted using SAS software (version 9.4 SAS Institute, Cary, NC, USA), and a significance level of *P* < 0.05 was applied for all tests.

## Results

### Growth performance

The results related to growth performance are presented in Table 3, Table 4. In the CRD analysis, no significant differences were observed in average daily weight gain between treatments up to day 10. In contrast, the Zn-ACT80 and Zn-ACT120 treatments exhibited significantly higher BWG during the period from 11 to 24 days of age compared to the control (zinc sulfate) and Zn-Gly40 treatments (*P* < 0.01). BWG over the entire period (0 to 42 days of age) was significantly increased in the Zn-Met80, Zn-ACT80, and Zn-ACT120 treatments compared to the control, Zn-Met40, and Zn-Gly40 treatments (*P* < 0.001). Feed intake did not differ significantly between experimental treatments across all periods (*P* > 0.05). During the period of 11 to 24 days of age, the Zn-Met80, Zn-ACT80, and Zn-ACT120 treatments exhibited improved FCR compared to the control and Zn-Gly40 treatments (*P* = 0.015). Similarly, during the entire 0- to 42-day period, the Zn-ACT80 and Zn-ACT120 treatments demonstrated better FCR values (1.69 and 1.70, respectively) than the Zn-Gly40 treatment (1.80) (*P* = 0.003). Although no significant differences were observed in mortality rates between treatments (*P* > 0.05), the uniformity rate increased during the 0- to 42-day period in chicks fed the Zn-ACT80 diet compared to the control and Zn-Met40 groups (*P* = 0.014). Regarding the EPI, the Zn-ACT80 and Zn-ACT120 treatments showed significantly higher EPI than the control and Zn-Gly40 treatments (*P* < 0.001). Additionally, the Zn-Met80 treatment showed a significant increase in EPI compared to the Zn-Gly40 treatment (*P* < 0.001).

**Table 3:** Effect of source and content of zinc (Zn) on body weight gain and feed intake of broiler chickens.

	Zn Source	Zn level (mg/kg)	Body weight gain (g/bird)				Feed intake (g/bird)			
Treatments			0-10 d	11-24 d	25-42 d	0-42 d	0-10 d	11-24 d	25-42 d	0-42 d
Control	Zn sulphate	120	203	818^b^	1,811	2,832^cd^	253	1,212	3,548	5,013
Zn-Met40	Zn-Met	40	204	851^ab^	1,791	2,847^cd^	254	1,213	3,548	5,014
Zn-Met80	Zn-Met	80	207	884^ab^	1,853	2,943^ab^	257	1,219	3,576	5,052
Zn-Met120	Zn-Met	120	206	866^ab^	1,845	2,916^abc^	257	1,208	3,567	5,032
Zn-Gly40	Zn-Gly	40	203	810^b^	1,801	2,813^d^	253	1,208	3,593	5,054
Zn-Gly80	Zn-Gly	80	203	838^ab^	1,852	2,893^abcd^	256	1,214	3,612	5,082
Zn-Gly120	Zn-Gly	120	204	859^ab^	1,857	2,920^abc^	260	1,216	3,589	5,065
Zn-ACT40	Zn-ACT	40	205	858^ab^	1,795	2,858^bcd^	249	1,212	3,561	5,022
Zn-ACT80	Zn-ACT	80	209	902^a^	1,863	2,974^a^	253	1,230	3,539	5,022
Zn-ACT120	Zn-ACT	120	207	898^a^	1,834	2,939^ab^	252	1,220	3,516	4,988
SEM			3.8	15.9	21.4	19.4	5.3	15.0	27.3	35.9
*P*-value: single factor (10 groups)^1^			0.981	0.002	0.123	<0.001	0.952	0.993	0.398	0.772
Main effect means										
Zinc source										
Zn-Met			206	867^ab^	1,830	2,902^ab^	256	1,213	3,564^ab^	5,033
Zn-Gly			203	836^b^	1,836	2,875^b^	256	1,213	3,598^a^	5,067
Zn-ACT			207	886^a^	1,831	2,924^a^	251	1,221	3,539^b^	5,011
SEM			2.3	9.4	12.6	10.7	3.1	8.9	15.6	20.7
Zinc level (mg/kg)										
40			204	840^b^	1,796	2,839^b^	252	1,211	3,567	5,030
80			206	875^a^	1,856	2,937^a^	255	1,221	3,576	5,052
120			206	874^a^	1,845	2,925^a^	256	1,215	3,557	5,029
SEM			2.3	9.4	12.6	10.7	3.1	8.9	15.6	20.7
*P*-value (3 × 3 factorial design)										
Zinc source			0.565	0.002	0.922	0.011	0.458	0.777	0.038	0.169
Zinc level			0.756	0.017	0.004	<0.001	0.585	0.722	0.712	0.671
Source × level			0.993	0.739	0.957	0.473	0.993	0.976	0.800	0.956

Values are means of 5 replicate pens per treatment and 28 male broiler chickens per replicate.

Means within each column series followed by different lowercase letters differ significantly (at *P* < 0.05).

1 Analysed as a completely randomized design. Abbreviations: Zn-Gly, zinc-glycine; Zn-Met, zinc-methionine; Zn-ACT, advanced chelate technology-based zinc.

**Table 4:** Effect of source and content of zinc (Zn) on feed conversion ratio (FCR), mortality rate (MR), uniformity rate (UR), and European performance index (EPI) of broiler chickens.

Treatments	Zn Source	Zn level (mg/kg)	FCR				MR (%)	UR (%)	
			0-10 d	11-24 d	25-42 d	0-42 d			EPI
Control	Zn sulphate	120	1.25	1.48^a^	1.96	1.77^ab^	5.71	89.9^b^	365^bc^
Zn-Met40	Zn-Met	40	1.24	1.43^ab^	1.98	1.76^ab^	3.57	89.5^b^	377^abc^
Zn-Met80	Zn-Met	80	1.24	1.38^b^	1.93	1.72^ab^	3.57	92.2^ab^	399^ab^
Zn-Met120	Zn-Met	120	1.25	1.40^ab^	1.93	1.73^ab^	4.29	93.3^ab^	391^abc^
Zn-Gly40	Zn-Gly	40	1.26	1.49^a^	2.00	1.80^a^	5.71	90.2^ab^	357^c^
Zn-Gly80	Zn-Gly	80	1.26	1.45^ab^	1.95	1.76^ab^	4.29	91.1^ab^	381^abc^
Zn-Gly120	Zn-Gly	120	1.27	1.42^ab^	1.93	1.73^ab^	5.00	92.1^ab^	386^abc^
Zn-ACT40	Zn-ACT	40	1.22	1.42^ab^	1.98	1.76^ab^	3.57	92.3^ab^	379^abc^
Zn-ACT80	Zn-ACT	80	1.21	1.37^b^	1.90	1.69^b^	3.57	94.7^a^	410^a^
Zn-ACT120	Zn-ACT	120	1.22	1.36^b^	1.92	1.70^b^	2.14	93.1^ab^	409^a^
SEM			0.036	0.028	0.028	0.018	1.083	1.00	7.5
*P*-value: single factor (10 groups)^1^			0.954	0.015	0.284	0.003	0.424	0.014	<0.001
Main effect means									
Zinc source									
Zn-Met			1.25	1.40^ab^	1.95	1.73^ab^	3.81	91.7^b^	389^ab^
Zn-Gly			1.27	1.45^a^	1.96	1.76^a^	5.00	91.1^b^	375^b^
Zn-ACT			1.22	1.38^b^	1.93	1.71^b^	3.10	93.4^a^	400^a^
SEM			0.021	0.017	0.016	0.010	0.637	0.56	4.3
Zinc level (mg/kg)									
40			1.24	1.45	1.99^a^	1.77^a^	4.29	90.7^b^	371^b^
80			1.24	1.40	1.93^b^	1.72^b^	3.81	92.7^a^	397^a^
120			1.25	1.39	1.93^b^	1.72^b^	3.81	92.8^a^	396^a^
SEM			0.021	0.017	0.016	0.010	0.637	0.56	4.3
*P*-value (3 × 3 factorial design)									
Zinc source			0.258	0.011	0.488	0.007	0.117	0.021	0.001
Zinc level			0.913	0.069	0.015	0.001	0.831	0.017	<0.001
Source × level			0.999	0.898	0.930	0.753	0.761	0.463	0.777

Values are means of 5 replicate pens per treatment and 28 male broiler chickens per replicate.

Means within each column series followed by different lowercase letters differ significantly (at *P* < 0.05).

1 Analysed as a completely randomized design. Abbreviations: Zn-Gly, zinc-glycine; Zn-Met, zinc-methionine; Zn-ACT, advanced chelate technology-based zinc.

In the factorial analysis, no significant interaction effects were observed between zinc source and dietary zinc level on growth performance parameters (*P* > 0.05). However, the Zn-ACT source significantly increased BWG (during 11 to 24 and 0 to 42 days of age) and EPI (0 to 42 days of age), while decreasing FCR (11 to 24 and 0 to 42 days of age) compared to the Zn-Gly source (*P* < 0.05). The Zn-Met source exhibited intermediate effects and did not differ significantly from the other sources (*P* > 0.05). The use of the Zn-ACT source also improved the uniformity rate compared to the Zn-Gly and Zn-Met sources. Furthermore, dietary zinc levels of 80 and 120 mg/kg significantly increased BWG (11 to 24, 25 to 42, and 0 to 42 days of age), uniformity rate (0 to 42 days), and EPI (0 to 42 days), while decreasing FCR (11 to 24 and 0 to 42 days) compared to the 40 mg/kg level (*P* < 0.05).

### Nutrient digestibility

The results related to nutrient digestibility are presented in Table 5. In the CRD analysis, no significant differences were observed in the digestibility of crude fat, ash, calcium, phosphorus, and energy between treatments (*P* > 0.05). In contrast, the ileal digestibility of crude protein was significantly higher in the Zn-Met80, Zn-Met120, Zn-ACT40, Zn-ACT80, and Zn-ACT120 treatments compared to the control and Zn-Gly40 treatments (*P* < 0.05). There was also a tendency for increased dry matter digestibility in treatments containing organic zinc supplements at levels of 80 and 120 mg/kg compared to the control treatment (*P* = 0.061).

**Table 5:** Effect of source and content of zinc (Zn) on nutrient digestibility in broiler chickens at 42 days of age.

			DM	CP	CF	Ash	Ca	P	Energy
Treatments	Zn Source	Zn level (mg/kg)				%			
1 (Control)	Zn sulphate	120	70.7	67.9^b^	77.8	49.4	41.9	53.9	73.4
2 (Zn-Met40)	Zn-Met	40	72.5	69.3^ab^	78.3	52.4	42.7	56.1	74.1
3 (Zn-Met80)	Zn-Met	80	73.9	70.5^a^	78.9	51.8	43.4	57.2	74.9
4 (Zn-Met120)	Zn-Met	120	73.2	69.7^a^	78.1	51.3	44.2	57.0	74.3
5 (Zn-Gly40)	Zn-Gly	40	71.9	67.9^b^	78.3	50.7	42.2	55.5	74.5
6 (Zn-Gly80)	Zn-Gly	80	73.0	68.5^ab^	78.5	51.6	43.1	56.2	74.6
7 (Zn-Gly120)	Zn-Gly	120	73.1	68.4^ab^	78.3	51.4	43.7	56.9	74.6
8 (Zn-ACT40)	Zn-ACT	40	72.3	70.2^a^	79.1	51.5	42.7	56.4	74.7
9 (Zn-ACT80)	Zn-ACT	80	74.0	70.3^a^	80.1	52.6	44.1	57.3	75.5
10 (Zn-ACT120)	Zn-ACT	120	73.4	70.4^a^	79.3	51.7	43.9	57.8	75.1
SEM			0.70	0.67	0.91	0.81	1.32	0.89	0.78
*P*-value: single factor (10 groups)^1^			0.061	0.028	0.817	0.289	0.948	0.174	0.861
Main effect means									
Zinc source									
Zn-Met			73.2	69.8^a^	78.4	51.8	43.4	56.7	74.4
Zn-Gly			72.7	68.3^b^	78.4	51.2	43.0	56.2	74.5
Zn-ACT			73.2	70.3^a^	79.5	52.0	43.6	57.1	75.1
SEM			0.41	0.40	0.54	0.47	0.76	0.52	0.46
Zinc level (mg/kg)									
40			72.2^b^	69.1	78.6	51.5	42.5	56.0	74.4
80			73.6^b^	69.8	79.2	52.0	43.5	56.9	75.0
120			73.2^a^	69.5	78.6	51.5	43.9	57.2	74.7
SEM			0.41	0.40	0.54	0.47	0.76	0.52	0.46
*P*-value (3 × 3 factorial design)									
Zinc source			0.553	0.003	0.264	0.533	0.864	0.468	0.567
Zinc level			0.050	0.520	0.656	0.655	0.427	0.226	0.699
Source × level			0.967	0.942	0.990	0.777	0.997	0.991	0.990

Values are means of 5 pens per treatment combination with 2 male broiler chickens. Means within each column series followed by different lowercase letters differ significantly (at *P* < 0.05).

1 Analysed as a completely randomized design. Abbreviations: Zn-Gly, zinc-glycine; Zn-Met, zinc-methionine; Zn-ACT, advanced chelate technology-based zinc; DM, dry matter; CP, crude protein, CF, crude fat; Ca, calcium; P, phosphorus.

In the factorial design analysis, the effects of dietary zinc source and level on the digestibility of crude fat, ash, calcium, phosphorus, and energy were not significant (*P* > 0.05). However, the zinc source had a significant effect on protein digestibility, with the Zn-Met and Zn-ACT treatments exhibiting significantly higher protein digestibility compared to the Zn-Gly source. Additionally, a significant effect of dietary zinc levels on dry matter digestibility was observed, with levels of 80 and 120 mg zinc/kg diet resulting in a significant increase in dry matter digestibility compared to 40 mg zinc/kg diet (*P* < 0.05).

### Serum antioxidant status

The results concerning blood antioxidant parameters are presented in Table 6. The CRD analysis showed that the Zn-Met80, Zn-Met120, Zn-Gly120, Zn-ACT80, and Zn-ACT120 treatments exhibited significantly lower serum MDA concentrations compared to the control, Zn-Met40, Zn-Gly40, and Zn-ACT40 treatments (*P* < 0.001). Additionally, serum TAC was significantly increased in the Zn-Met120, Zn-Gly120, Zn-ACT80, and Zn-ACT120 treatments compared to the control, Zn-Met40, and Zn-Gly40 treatments (*P* = 0.001). Serum SOD activity was elevated in chickens fed Zn-Met120 and Zn-ACT120 diets relative to the control, Zn-Met40, Zn-Gly40, and Zn-ACT40 groups (*P* < 0.001). Increased SOD activity was also observed in Zn-Met80, Zn-Gly120, and Zn-ACT80 treatments compared to the control and Zn-Gly40 treatments (*P* < 0.001). Serum CAT activity was significantly higher in the Zn-Met80 and Zn-ACT80 treatments compared to the control and Zn-Gly40 treatments (*P* = 0.001). The GPx activity was not significantly affected by the experimental treatments.

**Table 6:** Effect of source and content of zinc (Zn) on serum antioxidant status in broiler chickens at 42 days of age.

			MDA	TAC	GPx	SOD	CAT
Treatments	Zn Source	Zn level (mg/kg)	nmol/mL	mmol/L	U/mL	U/mL	U/mL
Control	Zn sulphate	120	2.91^a^	1.70^c^	917	123^c^	3.77^b^
Zn-Met40	Zn-Met	40	2.86^a^	1.76^c^	901	132^bc^	4.19^ab^
Zn-Met80	Zn-Met	80	2.04^bc^	2.27^abc^	1,013	169^ab^	5.31^a^
Zn-Met120	Zn-Met	120	1.95^bc^	2.52^a^	995	178^a^	5.07^ab^
Zn-Gly40	Zn-Gly	40	2.89^a^	1.74^c^	899	124^c^	3.73^b^
Zn-Gly80	Zn-Gly	80	2.42^abc^	2.15^abc^	1,011	142^abc^	4.11^ab^
Zn-Gly120	Zn-Gly	120	1.96^bc^	2.42^ab^	1,024	167^ab^	4.76^ab^
Zn-ACT40	Zn-ACT	40	2.74^ab^	1.83^bc^	926	134^bc^	4.58^ab^
Zn-ACT80	Zn-ACT	80	1.97^bc^	2.45^ab^	1,031	168^ab^	5.35^a^
Zn-ACT120	Zn-ACT	120	1.86^c^	2.56^a^	1,081	180^a^	5.17^ab^
SEM			0.167	0.206	68.4	9.0	0.316
*P*-value: single factor (10 groups)^1^			<0.001	0.009	0.555	<0.001	0.001
Main effect means							
Zinc source							
Zn-Met			2.28	2.18	970	160	4.86^a^
Zn-Gly			2.42	2.10	978	144	4.20^b^
Zn-ACT			2.19	2.28	1,013	161	5.04^a^
SEM			0.098	0.122	40.5	5.2	0.188
Zinc level (mg/kg)							
40			2.83^a^	1.78^b^	908	130^c^	4.17^b^
80			2.14^b^	2.29^a^	1,019	160^b^	4.93^a^
120			1.92^b^	2.50^a^	1,033	175^a^	5.00^a^
SEM			0.098	0.122	40.5	5.2	0.188
*P*-value (3 × 3 factorial design)							
Zinc source			0.251	0.594	0.731	0.056	0.009
Zinc level			<0.001	0.001	0.072	<0.001	0.005
Source × level			0.756	0.987	0.991	0.837	0.611

Values are means of 5 pens per treatment combination with 2 male broiler chickens. Means within each column series followed by different lowercase letters differ significantly (at *P* < 0.05).

1 Analysed as a completely randomized design. Abbreviations: Zn-Gly, zinc-glycine; Zn-Met, zinc-methionine; Zn-ACT, advanced chelate technology-based zinc; MDA, malondialdehyde; TAC, total antioxidant capacity; GPx, glutathione peroxidase; SOD, superoxide dismutase; CAT, catalase.

In the factorial analysis, no significant effects of zinc source or its interaction with dietary zinc level on serum antioxidant parameters were observed. In contrast, dietary zinc levels of 80 and 120 mg/kg significantly increased TAC, SOD activity, and CAT activity, while reducing MDA concentration compared to the 40 mg/kg zinc diet (*P* < 0.05). Furthermore, the highest SOD activity was observed at the 120 mg/kg zinc level (*P* < 0.05).

### Immune responses

The results pertaining to the relative weights of immune organs and humoral immune responses are presented in Table 7. In the CRD analysis, no significant effects of treatments were observed on the relative weights of the spleen, thymus, and bursa, nor on the secondary antibody titer against NDV (*P* > 0.05). In contrast, primary antibody titers against NDV and IBV (day 28) were significantly higher in the Zn-Met120, Zn-ACT80, and Zn-ACT120 treatments compared to the control treatment (*P* < 0.01). A tendency for increased secondary antibody titer against IBV (day 35) was also observed in treatments containing organic zinc supplements at levels of 80 and 120 mg/kg compared to the control (*P* = 0.087).

**Table 7:** Effect of source and content of zinc (Zn) on immune organ weights and antibody titers against Newcastle disease virus (NDV) and infectious bronchitis virus (IBV) in the serum of broiler chickens.

Treatments	Zn Source	Zn level (mg/kg)	Relative weight of immune organs (day 42)			Anti-NDV HI antibody titer, Log_2_		Anti-IBV ELISA antibody titer, Log_10_	
			Spleen	Thymus	Bursa	day 28	day 35	day 28	day 35
Control	Zn sulphate	120	0.119	0.260	0.103	2.41^b^	2.25	3.85^c^	3.58
Zn-Met40	Zn-Met	40	0.129	0.270	0.097	2.53^ab^	2.36	3.92^bc^	3.63
Zn-Met80	Zn-Met	80	0.138	0.284	0.114	3.06^a^	2.52	4.10^ab^	3.78
Zn-Met120	Zn-Met	120	0.125	0.290	0.115	2.98^ab^	2.46	4.07^abc^	3.77
Zn-Gly40	Zn-Gly	40	0.117	0.256	0.097	2.56^ab^	2.30	3.83^c^	3.55
Zn-Gly80	Zn-Gly	80	0.125	0.264	0.107	2.78^ab^	2.37	3.97^abc^	3.75
Zn-Gly120	Zn-Gly	120	0.130	0.259	0.118	2.91^ab^	2.42	4.03^abc^	3.74
Zn-ACT40	Zn-ACT	40	0.128	0.258	0.116	2.61^ab^	2.43	3.87^c^	3.64
Zn-ACT80	Zn-ACT	80	0.143	0.275	0.115	3.06^a^	2.61	4.10^a^	3.74
Zn-ACT120	Zn-ACT	120	0.145	0.292	0.119	3.02^a^	2.66	4.12^ab^	3.78
SEM			0.0082	0.0116	0.0106	0.123	0.110	0.047	0.064
*P*-value: single factor (10 groups)^1^			0.245	0.226	0.770	0.001	0.216	<0.001	0.087
Main effect means									
Zinc source									
Zn-Met			0.131	0.281	0.109	2.86	2.45	4.03^a^	3.73
Zn-Gly			0.124	0.260	0.107	2.75	2.36	3.94^b^	3.68
Zn-ACT			0.139	0.275	0.117	2.90	2.56	4.03^a^	3.72
SEM			0.0048	0.0064	0.0063	0.070	0.063	0.027	0.039
Zinc level (mg/kg)									
40			0.125	0.261	0.104	2.57^b^	2.36	3.87^b^	3.60^b^
80			0.135	0.274	0.112	2.97^a^	2.50	4.06^a^	3.75^a^
120			0.133	0.280	0.118	2.97^a^	2.51	4.08^a^	3.76^a^
SEM			0.0048	0.0064	0.0063	0.070	0.063	0.027	0.039
*P*-value (3 × 3 factorial design)									
Zinc source			0.111	0.059	0.523	0.332	0.090	0.041	0.653
Zinc level			0.270	0.118	0.301	<0.001	0.190	<0.001	0.010
Source × level			0.690	0.699	0.891	0.774	0.950	0.688	0.964

Values are means of 5 pens per treatment combination with 2 male broiler chickens. Means within each column series followed by different lowercase letters differ significantly (at *P* < 0.05).

1 Analysed as a completely randomized design. Abbreviations: Zn-Gly, zinc-glycine; Zn-Met, zinc-methionine; Zn-ACT, advanced chelate technology-based zinc; HI, hemagglutinin inhibition.

In the factorial design analysis, the effects of dietary zinc source and level on the relative weights of immune organs, as well as antibody titers against NDV and IBV, were not significant (*P* > 0.05). However, the zinc source significantly affected the primary antibody titer against IBV, with Zn-Met and Zn-ACT treatments showing significantly higher titers compared to the Zn-Gly source. Additionally, dietary zinc levels had a significant effect on the primary antibody titer against NDV, as well as primary and secondary titers against IBV; levels of 80 and 120 mg zinc/kg diet resulted in significantly higher antibody titers compared to 40 mg zinc/kg diet (*P* < 0.05).

### Zinc concentration in different tissues and zinc excretion

The results related to zinc concentrations in blood, tibia, liver, pancreas, and excreta are presented in Table 8. In the CRD analysis, serum zinc concentrations in the Zn-Met120, Zn-Gly120, and Zn-ACT120 treatments were significantly higher compared to the control, Zn-Met40, and Zn-Gly40 treatments (*P* = 0.001). Tibia zinc concentrations were significantly increased in the Zn-Met80, Zn-Met120, Zn-ACT80, and Zn-ACT120 treatments compared to Zn-Met40 and Zn-Gly40 treatments (*P* < 0.05), with the highest concentrations observed in the Zn-Met120 and Zn-ACT80 treatments. The highest pancreatic zinc concentrations were observed in the Zn-Met80 and Zn-ACT120 treatments, differing significantly from the control, Zn-Met40, Zn-Gly40, and Zn-ACT40 treatments (*P* < 0.05). Additionally, the Zn-ACT80 and Zn-Gly80 treatments exhibited higher pancreatic zinc concentrations compared to the Zn-Met40 treatment (*P* < 0.05). Zinc excretion was reduced in all experimental treatments except for the Zn-Gly120 group, with the lowest zinc levels in excreta observed in the Zn-Met80 and Zn-ACT40 treatments (*P* < 0.05). In contrast, liver zinc concentrations were not significantly affected by the experimental treatments.

**Table 8:** Effect of source and content of zinc (Zn) on Zn concentrations in different tissues and excreta of broiler chickens at 42 days of age.

			Serum	Tibia	Pancreas	Liver	Excreta
Treatments	Zn Source	Zn level (mg/kg)	µg/dL	mg/kg	mg/kg	mg/kg	mg/kg
1 (Control)	Zn sulphate	120	163^b^	222^abc^	67.0^bcd^	49.9	226^a^
2 (Zn-Met40)	Zn-Met	40	161^b^	215^cd^	63.0^d^	48.2	167^e^
3 (Zn-Met80)	Zn-Met	80	190^ab^	237^ab^	76.4^a^	50.6	179^cde^
4 (Zn-Met120)	Zn-Met	120	209^a^	240^a^	69.7^abcd^	54.7	198^bc^
5 (Zn-Gly40)	Zn-Gly	40	156^b^	198^d^	64.1^cd^	47.9	185^cde^
6 (Zn-Gly80)	Zn-Gly	80	186^ab^	221^abc^	72.5^abc^	50.9	195^bcd^
7 (Zn-Gly120)	Zn-Gly	120	195^a^	227^abc^	69.0^abcd^	51.5	209^ab^
8 (Zn-ACT40)	Zn-ACT	40	183^ab^	217^bcd^	65.3^cd^	48.1	175^de^
9 (Zn-ACT80)	Zn-ACT	80	188^ab^	239^a^	75.4^ab^	52.4	189^bcd^
10 (Zn-ACT120)	Zn-ACT	120	212^a^	236^ab^	76.2^a^	52.0	194^bcd^
SEM			9.3	6.4	2.67	1.75	6.7
*P*-value: single factor (10 groups)^1^			0.001	0.001	0.003	0.162	<0.001
Main effect means							
Zinc source							
Zn-Met			186	231^a^	69.7	51.2	181^b^
Zn-Gly			179	215^b^	68.5	50.1	196^a^
Zn-ACT			194	231^a^	72.3	50.8	186^b^
SEM			5.5	3.7	1.62	0.99	3.5
Zinc level (mg/kg)							
40			167^c^	210^b^	64.2^b^	48.1^b^	176^c^
80			188^b^	232^a^	74.8^a^	51.3^ab^	188^b^
120			205^a^	234^a^	71.6^a^	52.7^a^	200^a^
SEM			5.5	3.7	1.62	0.99	3.5
*P*-value (3 × 3 factorial design)							
Zinc source			0.163	0.007	0.251	0.746	0.015
Zinc level			<0.001	<0.001	0.000	0.007	<.0001
Source × level			0.659	0.954	0.643	0.716	0.810

Values are means of 5 pens per treatment combination with 2 male broiler chickens. Means within each column series followed by different lowercase letters differ significantly (at *P* < 0.05).

1 Analysed as a completely randomized design. Abbreviations: Zn-Gly, zinc-glycine; Zn-Met, zinc-methionine; Zn-ACT, advanced chelate technology-based zinc.

In the factorial analysis, no significant interaction effects of zinc source and dietary zinc level on zinc concentrations in different tissues were observed (*P* > 0.05). However, the Zn-Met and Zn-ACT sources increased zinc content in the tibia and decreased zinc excretion compared to the Zn-Gly source (*P* = 0.025). Dietary zinc levels of 80 and 120 mg/kg significantly increased serum, tibia, and pancreatic zinc concentrations compared to the 40 mg/kg treatment (*P* < 0.05). Furthermore, liver zinc concentrations were higher in the 120 mg/kg diet compared to the 40 mg/kg diet. Zinc excretion increased with increasing dietary zinc levels, with the highest excretion observed in the 120 mg/kg treatment (*P* < 0.05).

## Discussion

The findings of this study clearly demonstrate the positive effects of organic zinc sources, specifically Zn-ACT and Zn-Met, on the growth performance of broiler chickens. Significant improvements in BWG and FCR following supplementation with Zn-Met and Zn-ACT at 80 mg/kg indicate that these organic zinc sources possess greater bioavailability than inorganic alternatives such as ZnSO₄, resulting in enhanced absorption and utilization in broilers. These results align with previous research (Uniyal et al., 2017; Zakaria et al., 2017; De Grande et al., 2020) demonstrating that organic zinc sources promote the growth performance of broilers through improved nutrient absorption and metabolism. Notably, Xie et al. (2025) reported that organic zinc sources improve nutrient utilization efficiency without a corresponding increase in feed intake, corroborating the findings of the present study.

Furthermore, this experiment demonstrated that Zn-ACT and Zn-Met sources outperform Zn-Gly in enhancing growth performance. Chen et al. (2022) found that Zn-Met exhibited higher relative bioavailability than Zn-Gly in terms of metabolic zinc utilization, with approximately 1.20 to 1.25 times greater bioavailability as indicated by pancreatic metallothionein levels. Similarly, Ogbuewu and Mbajiorgu (2023) demonstrated that nano-zinc sources possess superior bioavailability compared to organic sources, largely due to their unique structural properties, which enhance mineral absorption. In a comparable manner, the Zn-ACT used in this study also benefits from a distinct structural advantage over other organic sources. Organic minerals produced using advanced chelation technology exhibit increased stability within the digestive tract, as the chelation structure resists gastric disintegration (Ghasemi et al., 2023; Mohammadizad et al., 2024). This enhanced stability may improve trace mineral absorption, thereby amplifying the beneficial effects on growth performance, feed conversion efficiency, and overall health. Beyond increased luminal stability, organic zinc sources may improve intestinal zinc uptake by up-regulating epithelial transport systems. In broilers, zinc proteinate of moderate chelation strength increased the expression of several zinc transporters (for example, ZnT1, ZnT4, ZnT7, ZnT9, ZnT10, ZIP3, and ZIP5) and amino-acid or peptide carriers relevant to chelate uptake (*y*+LAT2 and PepT1), compared with zinc sulfate, providing a mechanistic basis for higher portal zinc and improved bioavailability (Hu et al., 2022, 2023).

The enhanced uniformity rate observed in broilers supplemented with Zn-ACT and Zn-Met in this study also reflects the improved absorption and bioavailability of these zinc sources relative to Zn-Gly and ZnSO₄. The increase in flock uniformity associated with higher dietary zinc levels (80 and 120 mg/kg) further suggests that increased zinc supplementation promotes more consistent body weight gain, consistent with previous findings (Zhang et al., 2018; De Grande et al., 2020).

The results of this experiment indicate that both Zn-ACT and Zn-Met significantly enhance protein digestibility in broiler chickens. Increasing evidence suggests that the chemical form of dietary zinc substantially influences nutrient utilization efficiency. For example, Xie et al. (2019) reported that partially replacing inorganic zinc (ZnSO₄) with Zn-Met in the diets of weaned piglets improved nutrient digestibility, particularly when more than 50 % of inorganic zinc was substituted with organic zinc. Dong et al. (2023) also found that supplementation with zinc-chelated methionine hydroxy analogue (MHA-Zn) in low-protein diets enhanced protein digestibility, coinciding with an increase in beneficial gut microbiota such as *Lactobacillus* and *Butyricicoccus* species, which are critical for gut health and nutrient absorption. Although Kwiecień et al. (2017) reported beneficial effects of Zn-Gly on nutrient digestibility, the present study suggests that Zn-ACT and Zn-Met more effectively promote nutrient absorption. This may be attributed to their polymeric structures, which confer greater stability in the gastrointestinal tract, thereby enhancing mineral availability and reducing zinc loss during digestion. These observations indicate that the bioavailability of organic zinc is ligand-dependent; for example, Zn-Met showed greater bioavailability than Zn-Gly on corn–soybean diets, consistent with ligand-specific chelation stability and release properties (Hosseini et al., 2018; Xie et al., 2025). Zn-ACT is also characterized by its structural stability and controlled-release properties, enabling it to remain intact longer within the digestive tract, facilitating improved mineral absorption and contributing to the maintenance of gut health (Biabani et al., 2024a; Mohammadi et al., 2025). The study further revealed that higher dietary zinc levels (80 and 120 mg/kg) enhanced dry matter digestibility relative to the lower level of 40 mg/kg. This could be attributed to zinc’s cumulative action on the intestinal mucosa and its ability to stimulate digestive enzyme activity at elevated concentrations (Zarghi et al., 2022). Supporting these findings, Villagómez-Estrada et al. (2021) observed that supplementation of growing pigs with 80 mg/kg zinc hydroxychloride, compared to 20 mg/kg zinc sulfate, significantly improved nutrient digestibility. Overall, our results suggest that dietary supplementation with Zn-Met and Zn-ACT at 80 mg/kg effectively improves nutrient digestibility in broiler chickens, which could be a key factor contributing to enhanced growth performance in the respective groups.

Zinc plays a crucial role in the antioxidant defense system by assisting SOD in neutralizing reactive oxygen species (Arbabi-Motlagh et al., 2022). The findings of this study emphasize the importance of both the source and dosage of zinc in optimizing metabolic health and enhancing antioxidant defense. The increased antioxidant activity observed in birds fed Zn-Met and Zn-ACT supplements at levels of 80 and 120 mg/kg, as well as Zn-Gly supplementation at 120 mg/kg, was associated with higher SOD and CAT activities. This improvement in enzyme activity may contribute to more effective neutralization of reactive oxygen species. Additionally, these organic zinc sources resulted in higher TAC values and, consequently, reduced MDA levels, which serve as an oxidative stress marker. These results align with previous studies (Niknia et al., 2022; Yusof et al., 2023), which have shown that dietary supplementation with organic zinc increases antioxidant enzyme activity. Ghasemi et al. (2023) also demonstrated that advanced chelation technology improves antioxidant defense mechanisms by stabilizing trace minerals and thereby enhancing their bioavailability. Notably, the effects of Zn-ACT on antioxidant status were more similar to those of Zn-Met than to Zn-Gly, highlighting the superior bioavailability and effectiveness of Zn-ACT in enhancing antioxidant enzyme activity. These findings suggest that advanced chelate technology, as used in Zn-ACT, can enhance poultry health by improving zinc bioavailability, stabilizing trace minerals, and boosting antioxidant enzyme activity.

According to the results of the present study, dietary supplementation with Zn-Met at a dosage of 120 mg/kg and Zn-ACT at dosages of 80 and 120 mg/kg significantly enhanced the immune response in broilers, as evidenced by increased antibody titers against NDV and IBV vaccines. These findings are consistent with previous studies (Hosseini et al., 2018; Elsayed et al., 2021), which highlight the critical role of organic zinc sources in poultry immune function. Organic zinc sources exhibit greater bioavailability than their inorganic counterparts, leading to enhanced immunogenic effects (Saleh et al., 2018; Bortoluzzi et al., 2019). Al-Mayah and Al-Mashhadani (2025) also reported that broilers supplemented with 90 mg/kg of Zn-Met showed significantly improved antibody responses to NDV, which were attributed to enhanced zinc uptake and retention through its combination with methionine. Furthermore, advanced chelate structures, which improve mineral stability and absorption by forming stable complexes with organic acids, have also been shown to promote positive immune responses in broilers (Biabani et al., 2024b). The mechanisms contributing to the improved antibody responses induced by vaccines are primarily attributed to the enhanced bioavailability of organic zinc, which ensures sufficient systemic availability for optimal immune cell function (Chand et al., 2020). Additionally, zinc plays a crucial role in the antioxidant defense system as a cofactor for SOD, which neutralizes reactive oxygen species and mitigate oxidative stress that could compromise immune function (Saleh et al., 2018; Mohammadizad et al., 2024). Moreover, zinc plays an essential role in regulating gene expression associated with immune responses, particularly genes that control cytokine production and lymphocyte proliferation (Bortoluzzi et al., 2019). These regulatory pathways facilitate the optimal production of antibodies following vaccination.

The findings of this study align with previous research suggesting the relative bioavailability advantages of organic zinc supplements over their inorganic counterparts. Organic zinc forms have been shown to improve bone quality (Min et al., 2019), increase tissue zinc concentration (Chen et al., 2022), and reduce fecal zinc excretion (Bao et al., 2007) compared to zinc sulfate. In the present study, comparisons between different zinc sources and their supplementation levels revealed that the effects on zinc storage in various tissues depend not only on the type of zinc source but also on the level of supplementation. Among the organic sources, Zn-Met and Zn-ACT at dosages of 80 and 120 mg/kg were more effective than other treatments. These results suggest that the optimal supplementation level for maximizing bioavailability in Zn-Met and Zn-ACT is 80 mg/kg. Zn-Met contains methionine, a sulfur-containing ligand that offers high stability in the digestive tract. This stability prevents zinc from undergoing negative interactions with other substances, thereby enhancing its absorption (Mohamed et al., 2023). The results of this study are consistent with those of Mwangi et al. (2017), who demonstrated that substituting inorganic zinc with Zn-Met improved feed efficiency, boosted tissue zinc storage, and reduced zinc excretion in chickens. Similarly, Singh et al. (2015) observed a significant reduction in zinc excretion following supplementation with methionine chelates. Further research supports the role of the physicochemical structure of zinc supplements in zinc absorption and retention. Wu et al. (2019) demonstrated that supplementation with micronized zinc, which has a high specific surface area, increased pancreatic zinc concentrations and improved antioxidant activity in ducks. The stable, polymeric structure of advanced chelate supplements emphasizes the importance of the physicochemical stability of zinc supplements in determining their efficacy (Ghasemi et al., 2020, 2023; Mohammadizad et al., 2024).

## Conclusions

Based on the results of this study, Zn-Met and Zn-ACT consistently outperformed Zn-Gly and ZnSO₄ in growth performance, feed efficiency, antioxidant status, immune indices, and indices of zinc utilization. At 80 mg/kg diet, Zn-ACT achieved outcomes comparable to, and in several cases greater than, the 120 mg/kg level, indicating high bioavailability at a lower inclusion rate. Both Zn-Met and Zn-ACT reduced fecal zinc excretion, suggesting a lower environmental zinc load relative to Zn-Gly and ZnSO₄. When directly compared with Zn-Met, Zn-ACT improved flock uniformity from day 0 to day 42, while other responses were similar between these two organic sources. Collectively, the results support Zn-ACT, particularly at 80 mg/kg diet, as an effective strategy for enhancing broiler performance and health while mitigating zinc excretion.

## CRediT authorship contribution statement

**Mousa Nasiri:** Writing – original draft, Validation, Investigation, Data curation, Conceptualization. **Hossein Ali Ghasemi:** Writing – review & editing, Writing – original draft, Supervision, Methodology, Investigation, Formal analysis. **Iman Hajkhodadadi:** Writing – original draft, Visualization, Software, Resources, Methodology. **Mohammad Hassan Nazaran:** Writing – review & editing, Writing – original draft, Validation, Resources, Project administration, Conceptualization.

## Disclosures

All authors approve the submission of this manuscript and declare no conflict of interest.
